# Delactylase effects of SIRT3 on a positive feedback loop involving the RUNX1-glycolysis-histone lactylation in diabetic kidney disease

**DOI:** 10.7150/ijbs.126011

**Published:** 2026-01-15

**Authors:** Siman Shen, Chen Ying, Xinglin Fu, Xiaobian Zeng, Xiuli Guo, Han Wu, Liangqing Zhang, Li Xu

**Affiliations:** 1 Department of Anesthesiology, The Second Affiliated Hospital of Guangdong Medical University, Zhanjiang, Guangdong 524003, China.; 2 Department of Nephrology, The First Hospital of China Medical University, Shenyang, Liaoning 110001, China.; 3 Department of Laboratory Medicine, The Second Affiliated Hospital of Guangdong Medical University, Zhanjiang, Guangdong 524003, China.

**Keywords:** diabetic kidney disease, tubular epithelial cells, SIRT3, histone lactylation

## Abstract

**Background:** Persistently elevated glycolysis is increasingly recognized as a driving force in diabetic kidney disease (DKD). As a product of glycolysis, lactate can induce histone lactylation, an emerging epigenetic mechanism associated with post-transcriptional modification. However, the molecular mechanism and clinical impact of histone lactylation in DKD remain largely understood.

**Methods and Results:** Spatial transcriptomics analysis revealed upregulation of glycolytic genes in tubular epithelial cells (TECs), thus leading to elevated levels of renal lactate accumulation. PKM2 deficiency lowered the lactate production during the fibrotic process and decreased histone lactylation. Mechanistically, ChIP-seq & RNA-seq results showed lactate promoted histone H4 lysine 12 lactylation (H4K12la), which in turn enhanced RUNX1 transcription. RUNX1 subsequently activated HK1 and SLC2A1, which accelerated glycolysis and renal fibrosis of DKD. Further, SIRT3 expression was significantly decreased in the renal tubular cells in DKD. Furthermore, insufficient SIRT3 is functionally promote renal fibrosis by directly deacetylating RUNX1 at H4K12, leading to attenuated glycolytic process, and subsequently robust glycolytic ability and increased production of lactate.

**Conclusion:** Thus, the study links RUNX1-mediated glycolysis to SIRT3-mediated histonelactylation epigenetic reprogramming in promoting the fibrotic process, providing better understanding of epigenetic regulation of DKD pathogenesis, and new therapeutic strategy for DKD.

## 1. Introduction

Diabetes is a primary cause of chronic kidney disease (CKD), with up to 40% of individuals with diabetes developing CKD. Between 1990 and 2017, the incidence of CKD among people with type 2 diabetes increased by 74% 1. Hyperglycemia initiates a cascade of molecular changes, resulting in heightened oxidative stress and an elevation in inflammatory cytokines, growth factors, and profibrotic substances 2, 3. This imbalance leads to structural and functional disruptions at both the molecular and cellular levels, contributing to mesangial expansion, podocyte injury, tubulointerstitial fibrosis, and glomerulosclerosis. Eventually, these metabolic disturbances further damage kidney function and impair homeostasis 4, 5. Tubular epithelial cells (TECs) exhibit heightened vulnerability to injury. In diabetic kidney disease (DKD), impaired or insufficient regeneration of TECs is a hallmark feature and has been recognized as a major contributor to renal inflammation and fibrosis 6-8. Nevertheless, the mechanisms underlying TEC injury and the progression of renal fibrosis remain largely unknown.

TECs are rich in mitochondria, making them more sensitive to hypoxia in stressful environments compared to other intrinsic kidney cells 9, 10. In pathological conditions, TECs are prone to mitochondrial dysfunction, leading to metabolic imbalance. Under pathological condition, the expression of key glycolytic enzymes is upregulated in damaged TECs, converting the energy metabolism pathway from FAO to glycolysis 11, 12. Although compensatory glycolysis can partially reverse the energy imbalance, prolonged aerobic glycolysis can lead to the accumulation of metabolic intermediates, especially lactate 13. Which triggers the release of inflammatory factors, exacerbates mitochondrial damage, induces epithelial-mesenchymal transition (EMT) in tubular epithelial cells (TECs), and promotes renal interstitial fibrosis 14-16.​ Lactate, derived from glycolysis, has been recognized as a substrate for histone lactylation, directly activating downstream gene transcription 17, 18. Altered glycolysis and gluconeogenesis in kidneys significantly disturb the lactate metabolic balance, which exert impacts on the severity and prognosis of kidney injury 19. Notably, histone lactylation serves as an important epigenetic regulatory mechanism in renal disease pathogenesis. For instance, lactate produced by Lgals3 promoted lactylation at H3K18, which enhanced FGFR4 expression and contributes to the formation of calcium oxalate (CaOx) kidney stones and renal fibrosis 20. Furthermore, the overproduction of lactate induced by PKM2 facilitates H3K18 lactylation, which subsequently activates the Smad3 signaling pathway in renal tubular cells, thereby driving renal fibrosis 21. Recent study has found lactate increased the levels of H3K14la that attributed to the EMT progression and renal tubular fibrosis in DKD 22. The exact role of histone lactylation in the progression of DKD remains largely unknown. Investigating PTMs in TECs may help elucidate the underlying mechanisms regulating TEC injury in DKD.

SIRT3, a member of the Sirtuin family, is a highly conserved NAD+-dependent lysine deacylase that removes acyl modifications from cellular substrates. SIRT3 plays a crucial role in regulating genomic stability, metabolic balance, stress responses, and aging 23, 24. SIRT3 knockout or silencing could significantly aggravate mitochondrial injury and suppressed mitophagy in diabetic cardiomyopaphy 25. Moreover, SZC-6 directly binds to SIRT3 and enhances its deacetylating activity on SIRT3, restores mitochondrial function, significantly alleviates the pathological damage of DKD 26, indicating that targeted activation of SIRT3 could be a promising therapeutic strategy for DKD. Additionally, recent discoveries identify SIRT3 as a dual-function regulator of histone lactylation, possessing delactylase activity and metabolic control over lactate pools 27. Thus, it is hypothesis that SIRT3 serves as a molecular rheostat coordinating lactylation-dependent kidney fibrosis in DKD.

Therefore, we uncovered novel functions of SIRT3 in regulating histone lactylation, particularly in H4K12la levels, and revealed a positive feedback loop involving RUNX1 and glycolysis, driven by histone lactylation. Furthermore, we propose that this feedback loop represents a potential therapeutic target for DKD.

## 2. Materials and Methods

### 2.1 Human specimens

The renal biopsies samples of DKD were obtained from Department of Nephrology, the First Hospital of China Medical University with needle biopsies. Normal control samples were obtained from healthy kidney tissues of tumor nephrectomy samples with wedge biopsy. Our studies were approved by the Clinical Research Ethics Committee of the First Hospital of China Medical University and were conducted in accordance with the Declaration of Helsinki principles.

### 2.2 Animal experimental design

The db/db mice, along with their lean littermate counterparts of the same age (db/m) (BKS.Cg-Dock7m^+/+^ Leprdb/J), were acquired from the Model Animal Research Center at Nanjing University. Approval for all animal experiments was granted by the Ethics Committee regarding the Care and Use of Laboratory Animals at Guangdong Medical University. The animals were housed in a controlled environment with a standard light/dark cycle and maintained under specific pathogen-free (SPF) conditions. Mice in the treatment groups received intraperitoneal injections of lactate (0.5 g/kg body weight, pH adjusted to 6.8) for 10 weeks. Additionally, FX-11 (15 mg/kg) was administered intraperitoneally for 10 weeks.

The shRUNX1 packaged in an adeno-associated virus (AAV) was sourced from GeneChem Company (Shanghai, China) and was injected directly into the renal pelvis of the db/db group. Our study was carried out after approving by the Ethics Committee on the Care and Use of Laboratory Animals of Guangdong Medical University (registration number: No. GDY2402166, 27th Febrary 2024).

### 2.3 Biochemical analysis

At eight weeks, blood samples were collected from the tail vein of each group for biochemical evaluation. A blood glucose concentration surpassing 16.7 mM validated the effective creation of the diabetic mice model. Blood glucose (Roche Diabetes Care, IN, USA), urine creatinine, and urine albumin levels (Roche Diagnostics, IN, USA) were measured every 4 weeks, following the methods described previously 28. Starting from 10 weeks, AAV-shRUNX1 was administered, and all groups were fasted for a minimum of 8 hours prior to blood glucose and urine tests, with water allowed during the fasting period. Following euthanasia at 20 weeks, blood and tissue specimens were harvested.

### 2.4 Cell culture and transfection

The HK-2 human renal tubular epithelial cell line, sourced from ATCC (MD, USA), was cultured in DMEM/F12 medium containing 10% fetal bovine serum (FBS), with medium changes occurring every 72 hours. For the experiments, the cells were categorized into NG and HG (30mmol/L) groups. For the indicated experiments, HK-2 cells were treated with sodium lactate (La) (cat. no. S108838, Aladdin), lactate dehydrogenase inhibitor (FX-11, Aladdin), HDAC inhibitor (TSA, GC15526, GLPBio), P300 inhibitor (C646, ab142163, Abcam) and Sirtuins inhibitor (NAM, HY- B0150, MedChemExpress).

The overexpression plasmid for RUNX1, SIRT1-3, and SIRT5-7 were generated by amplifying the corresponding genes and inserting them into the pcDNA3.1 eukaryotic expression vector through subcloning.

### 2.5 Gathering and analyzing spatial transcriptomics data of DKD patients

The ST data for the diabetic kidney disease (DKD) human kidney sample has been deposited in the Gene Expression Omnibus (GEO) at the National Center for Biotechnology Information (https://www.ncbi.nlm.nih.gov/geo/query/acc.cgi?acc=GSE261545). The ST datasets used in this study were obtained from the GEO repository (accession number: GSE261545).

The data underwent dimensionality reduction utilizing the Seurat package (version 4.2.2) within the R programming environment. For the purposes of quality control, spatial spots containing a minimum of five unique genes or unique molecule identifiers were preserved in the Seurat object. The clustering based on morphology that had been previously established was integrated into the metadata. SCTransform was employed for data normalization and scaling, and the top 3000 variable features were identified for principal component analysis. The initial 20 principal components were utilized in Uniform Manifold Approximation and Projection (UMAP) to achieve dimensionality reduction, resulting in an equivalent number of clusters for comparison against the morphology-based clusters. Subsequently, high-resolution spatial plots were generated to illustrate the localization of these clusters along with the expression levels of the feature genes.

The Loupe file generated by the 10x Space Ranger pipeline was first accessed using the Loupe Browser. A kidney pathologist (P.I.) utilized H&E-stained bright-field mosaics (ST slide) as references to conduct morphology-based clustering for every spatially resolved spot on the Visium slide. This procedure entailed manually delineating the morphological structures and lesions of the kidney to recognize the clusters.

### 2.6 Histology and immunohistochemistry (IHC)

Renal tissues from both humans and mice were sectioned into 3 μm thickness. The samples underwent staining using Masson, Sirius red, and periodic acid-Schiff (PAS) methods before being examined under a microscope. Immunohistochemical analyses (IHC) were performed in accordance with the manufacturer's instructions. Specific primary antibodies targeting PKM2 (NBP1-48308, Novus Biologicals), HK2 (ab104836, Abcam), αSMA (ab5694, Abcam), Vimentin (ab92547, Abcam), FN (ab2413, Abcam), NF-κB p65 (sc-8008, Santa Cruz Biotechnology), RUNX1 (ab23980, Abcam) were incubated overnight at 4 °C. The samples were then treated with secondary antibodies for one hour at room temperature. Afterward, they were stained using diaminobenzidine (DAB), counterstained with hematoxylin, and observed under a microscope.

### 2.7 Lactate, glucose and ATP measurement

Lactate levels in renal tissue and HK-2 cells were quantified using the Lactate Colorimetric Assay Kit (Biovision, K627-100) at 450 nm. Glucose concentrations in the cell supernatant were measured with the Glucose Colorimetric/Fluorometric Assay Kit (Biovision, K606-100), also at 450 nm. ATP concentrations were assessed with a commercially available ATP colorimetric assay kit (K354-100, BioVision). In summary, samples of fresh tissue or cell suspensions were lysed and subjected to centrifugation at 12,000 rpm for 5 minutes at a temperature of 4 °C. Eventually, 20 μL of the supernatant was combined with 100 μL of luciferase reagent, and ATP levels were measured using a microplate luminometer (Promega, Madison, WI, USA).

### 2.8 Immunofluorescence (IF)

Human and mice kidney tissue sections (3 µm thick) were frozen and prepared. For fixation, HK-2 cells were incubated with cold methanol/acetone solution at room temperature for 10 minutes, then incubated with 10% donkey serum for 1 hour to block non-specific binding. Primary antibodies targeting E-cadherin (sc-59778, Santa Cruz Biotechnology), PKM2(NBP1-48308, Novus Biologicals) H4K12la (PTM-1411RM, PTMBio), RUNX1 (sc-365644, Santa Cruz Biotechnology), HK1 (ab150423, Abcam), SLC2A1 (MAB1418, R&D Systems), FN (sc-8422, Santa Cruz Biotechnology), αSMA (NB300-978, Novus Biologicals) then incubated with the secondary antibodies for one hour. 4,6-diamidino-2phenylindole (DAPI, Sigma-Aldrich) was used as a counterstain for 10 minutes on the nuclei.

### 2.9 Western blot

Mice kidney tissues and HK-2 cells were used to extract protein. Equal amounts of each sample (20 μg) underwent separation using 10% SDS-PAGE, followed by transfer to PVDF membranes. The following primary antibodies were applied: RUNX1 (ab23980, Abcam), αSMA (ab5694, Abcam), Vimentin (ab92547, Abcam), FN (ab2413, Abcam), Pan Kla (PTM1401, PTMBio), H4K12la (PTM-1411RM, PTMBio), Histone H4 (ab177840, Abcam), HK1 (ab150423, Abcam), SLC2A1 (ab115730, Abcam), SIRT3 (ab118334, Abcam) (all at a 1:1000). β-tubulin (1:10000) was used as a reference control. Subsequently, the secondary antibodies (1:10,000, Novus Biologicals) were applied to the membranes and left to incubate for 2 hours at room temperature. Band densities were analyzed and quantified using ImageJ software.

### 2.10 RT‒qPCR

The renal tissue and HK-2 cells were extracted total RNA using Tizol reagents (Takara, Kusatsu, Japan). The RNA concentration was examined using a Nanodrop system (ThermoFisher Scientific). RT‒qPCR was performed with SYBR Green PCR Master Mix and analyzed using a Roche RT-PCR System. β-actin was used as a negative control. 2^-ΔΔCT^ method was performed to assess the relative expression of mRNA. All the PCR analysis were conducted out following MIQE guidelines. Primers used in this study were listed in Supplementary Table S1.

### 2.11 CUT&Tag assay

HK-2 cells were isolated, counted, and centrifuged at 600×g for 3 minutes at room temperature. Cells totaling 1 × 10^5^ were gently pipetted and washed twice with 500 μL of wash buffer. Magnetic beads that were coated with Concanavalin A were prepared as per the instructions provided in the kit, and then 10 μL of the activated beads were incorporated into each sample, which was incubated at room temperature for 10 minutes thereafter. Following the kit instructions, cells were incubated sequentially with ConA beads, primary antibody (anti-H4K12la antibody, PTM-1411RM, PTMBio), secondary antibody (Goat anti-Rabbit IgG H&L, AB206-01-AA), and Hyperactive PG-TN5 / PA-TN5 transposon, followed by fragmentation. The fragmented DNA was then extracted and amplified by PCR. The generation of CUT&TAG libraries was accomplished using the CUT&Tag-seq kit (904, Nanjing Vazyme Biotech Co., Ltd., Nanjing, China). The quality of the library was assessed using LabChip Touch, and sequencing was performed on an Illumina NovaSeq platform. The sequencing data were subsequently analyzed by SeqHealth Tech Co. Ltd (Wuhan).

### 2.12 ChIP-qPCR assay

ChIP was carried out utilizing the Chromatin Immunoprecipitation Kit from Millipore (17-371). Subsequently, the precleared chromatin was allowed to incubate overnight at 4 °C with either an H4K12la antibody (PTM Bio, PTM-1411) or a control IgG during the immunoprecipitation procedure. ChIP-enriched DNA was investigated by qPCR with promoter-specific primers and ChIP DNA enrichment was determined by % Input and then represented as fold-over IgG. The precipitated chromatin DNA was purified and detected by qPCR using specific primers shown in Table S2.

### 2.13 RNA-sequencing and analysis for differential expressed transcripts

Total RNA was isolated using Trizol (Invitrogen, Carlsbad, CA, USA). RNA sequencing analysis was conducted with technical support from Aksomics Biotech Co. Ltd (Shanghai, China). Differentially expressed genes were determined based on raw p-values and fold changes. Genes with a corrected p-value < 0.05 and an absolute fold change > 2 in multiple tests were considered significantly differentially expressed.

### 2.14 Extracellular acidification rate (ECAR) and Mitochondrial oxygen consumption rate (OCR) measurement

The Seahorse Bioscience extracellular flux analyzer was utilized to measure ECAR and OCR. For this experiment, cells were grown in XF24 V7 cell culture microplates (Seahorse Bioscience) under conditions of 10 mM glucose for 2 hours at 37 °C. Subsequently, either the ATP synthase inhibitor oligomycin or the glycolysis inhibitor 2-deoxyglucose (2-DG) was introduced, and the findings were assessed as ECAR. Alternatively, cells underwent treatment with oligomycin (1 μM), FCCP (0.5 μM), or a combination of antimycin A (0.5 μM) along with rotenone (0.5 μM), and the measurements were taken, with the results expressed as OCR values.

### 2.15 NAD+ measurement

20 mg of kidney tissue was homogenized in extraction buffer provided by the kit (E2ND-100, Bioassay systems) and NAD+ was determined immediately according to manufacturer's protocol.

### 2.16 Molecular docking

In this study, the Histone4 structure was predicted using the AlphaFold3 model, with the Uniprot database ID being B4E380. Lactation modification was performed on Lys194 (K14) using UCSF Chimera. The crystal structure of SIRT3 protein was obtained from the PBD database, with the number 3GLR. Small molecules and other heteroatoms were removed while the protein structure was retained. The three-dimensional structure of the protein was optimized using UCSF Chimera, and the atomic charge of AMBER14SB was assigned. The PKa value of amino acids and the allocation of hydrogen atoms under neutral conditions were calculated using the H++3 online tool. Then, HDOCK was used for molecular docking. The empirical iterative scoring function ITScoreNP was employed for global search and scoring of molecular conformations. Among the various docking configurations, the configuration that exhibited the highest docking and confidence scores was chosen for further analysis. For the 3D plotting analysis, PyMOL2.04 was utilized.

### 2.17 Co-immunoprecipitation (CO-IP) assay

Total protein from the cell lysate was subjected to immunoprecipitation. The extract was first incubated with anti-SIRT3 (sc-365175, Santa Cruz Biotechnology) for 24 hours at 4 °C. A mixture was supplemented with Protein A/G Agarose and incubated for another 3 hours at 4 °C. Following this, centrifugation was performed at 12,000 rpm for 5 minutes to collect the precipitate. Afterward, the precipitate was washed, resuspended in 40 μL of SDS lysis buffer, and boiled for 5 minutes. Ultimately, the precipitate was subjected to immunoblot analysis utilizing the designated antibody.

### 2.18 Statistical analysis

Quantitative data were obtained from independent experiments with three replicates and are presented as the mean ± SD. Statistical analysis was performed using SPSS 15.0 software (SPSS Inc., Chicago, IL, USA). Comparisons between the two groups utilized an unpaired Student's t-test, which was two-tailed, while multiple comparisons were assessed using a one-way ANOVA with a Bonferroni correction. A P-value below 0.05 was regarded as statistically significant.

## 3. Results

### 3.1 Elevated glycolysis mediated lactate accumulation is correlated with renal dysfunction in DKD patients

**Figure 1:**
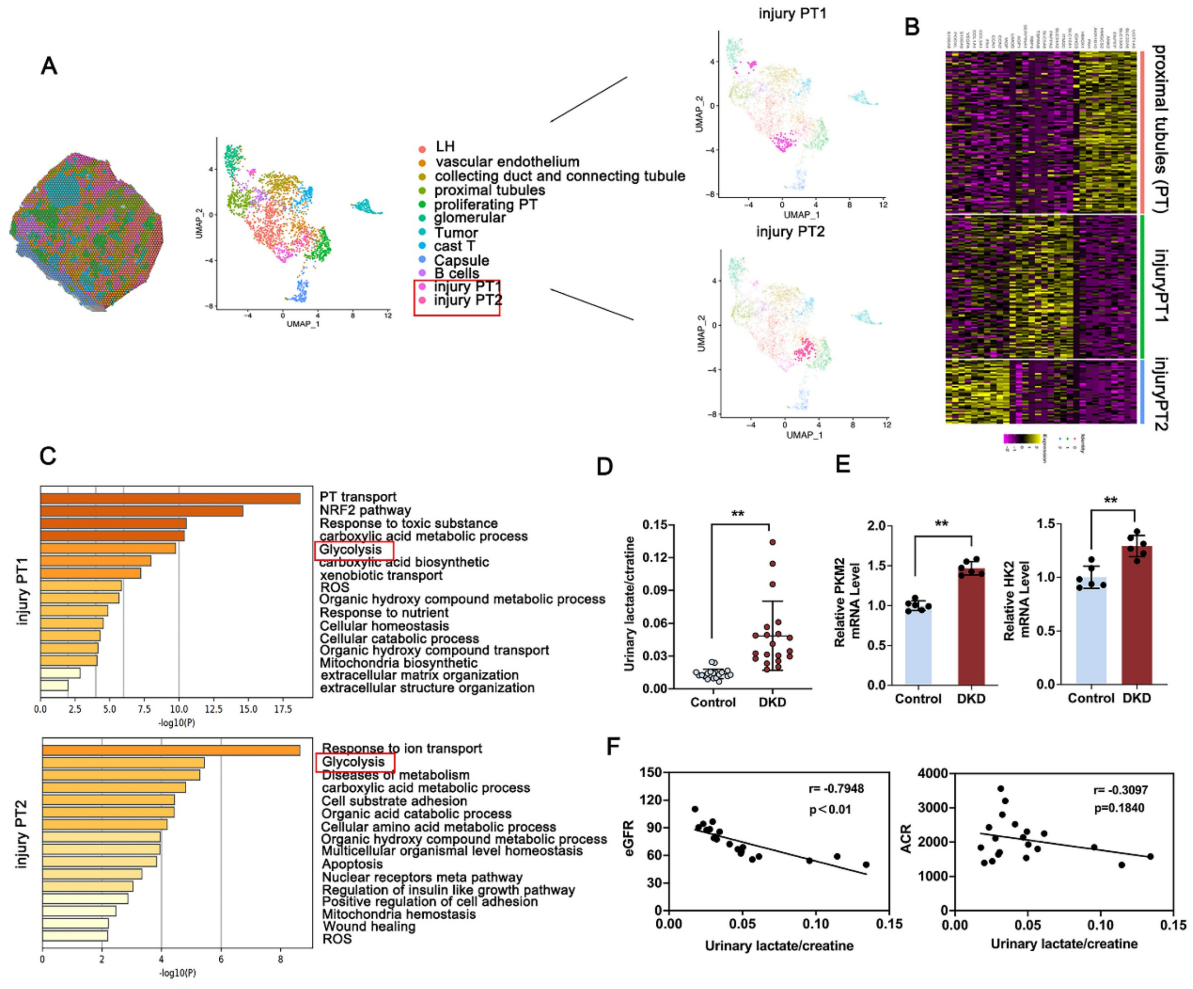
** Activation of glycolysis and PKM2 expression attributed to injury proximal tubules in DKD.** A. Spatial distribution and Uniform Manifold Approximation and Projection (UMAP) analysis of clusters derived from pathological classification; B. Heatmap of differentially expressed genes between PT, injury PT1 and injury PT2 subcluster; C. Gene Ontology Biological Process (GO-BP) enrichment analysis of subcluster-specific genes in injured proximal tubule (PT) cells; D. Comparison of urinary lactate/creatinine ratios between healthy individuals and patients with DKD (n=20); E. RT-qPCR analysis for renal PKM2 and HK2 in Control and DKD patients (n=6); F. Analysis of the correlation between urinary lactate/creatinine levels and eGFR (B) or ACR (C) in DKD patients. Data are presented as mean ± standard deviation from three independent experiments. **P<.01versus Control group by Student's t-test.

To analyze the tubular compartment in DKD kidney for further facilitating the possible identification of tubular lesion, bioinformatics analysis was performed using spatial transcriptomics (ST) data from kidney obtained from DKD patients 29. Morphologic analysis revealed 12 discrete compartments and lesion types, including sections of loop of Henle ducts, vascular endothelium, collecting duct and connecting tubule, proximal tubule (PT), proliferating PT, glomerular, tumor, cast T, capsule, B cells, and the subcluster analysis of the PT cluster into injury PT1 and injury PT2 (Figure 1A). Notably, clusters based on PT and injury-related PT exhibited distinct and substantial expression enrichment of recognized marker genes (Figure 1B). GO-based pathway analysis, contrasting the PT cluster with the injury-associated PT1 or PT2 cluster, highlighted the enrichment of glycolysis-related pathways, which are known contributors to DKD progression (Figure 1C). Lactate is known as a product of aerobic glycolysis contribute to the progression of fibrosis across multiple diseases 30, 31. To investigated the clinical relevance of lactate accumulation in DKD patients, we initially observed that urinary lactate-to-creatinine levels were markedly elevated in DKD patients (Figure 1D), showing an inverse relationship with eGFR, but no significant association with urine albumin-to-creatinine ratio (ACR) (Figure 1F). Elevated induction of glycolysis-related gene expression, including HK2 and PKM2, in the kidneys was further confirmed in Figure 1E. Further, Immunohistochemistry (IHC) staining of PKM2 and HK2 were used to determine the localization of HK2 and PKM2 expression and indicated a noticeable increase in both DKD patients (Figure S1A). To further investigate PKM2 distribution, we conducted immunofluorescence co-localization staining using an antibody against E-cadherin, a marker for renal tubular epithelial cells. The findings revealed that PKM2 was primarily upregulated in TECs, with negligible expression observed in glomerular endothelial cells and podocytes of db/db mice and DKD patients (Figure S1B). Altogether, metabolic abnormality facilitated activated glycolysis pathway, leads the production of lactate and renal dysfunction in DKD.

### 3.2 PKM2-driven lactate accumulation promotes renal fibrosis in DKD

To clarify whether excessive lactate accelerate renal tubular fibrosis in DKD, the db/db mice was administrated with supplemental lactate or FX-11 (a specific inhibitor of lactate dehydrogenase) for 10 weeks (Figure S2A and S3A). The results indicated that lactate supplementation markedly elevated renal lactate levels and FX-11 suppressed renal lactate accumulation in comparison to db/db mice (Figure S2B and S3B). Masson and Sirius red staining revealed that lactate administration accelerated pathological damage, however, attenuation following FX-11 administration compared with db/db group (Figure S2C and S3C). Lactate administration exhibited an increased expression of αSMA, Vimentin and FN compared with db/m group, and excessive lactate production aggravated the fibrotic levels but reversed with FX-11 administration compared with db/db group (Figure S2C-D and S3C-D).

PKM2 display the highest kinase-to-phosphatase ratio, facilitating the shift of glucose toward glycolysis. PKM2 inhibition markedly reduced lactate production of HK-2 cells in contrast to the control group (Figure S4A). Furthermore, renal tubular epithelial cells (HK-2) transfected with siPKM2 plasmid resulted in decreased expression of FN and α-SMA compared with the control group (Figure S4B-C). As shown in Figure S4D, 2/9 AAV vector of knockdown PKM2 or control vector was injected into the renal pelvis of db/db mice. Consistent with the results in vitro, IHC, Sirius red and MASSON staining revealed that the accumulation of ECM and the expression of FN and αSMA were reversed by silencing of PKM2 compared with db/db group (Figure S4E). The expression of αSMA and FN were further confirmed by Western blot in Figure S4G. Besides, the expression of simultaneous down-regulation of FN and E-cadherin indicated fibrosis process was alleviated with silencing of PKM2 compared with db/db group in Figure S4F. These findings suggest that targeting tubular PFKFB3 may directly inhibit the accumulation of lactate, thus alleviating fibrosis process in DKD.

### 3.3 PKM2-driven lactate accumulation promotes histone lactylation in DKD

**Figure 2:**
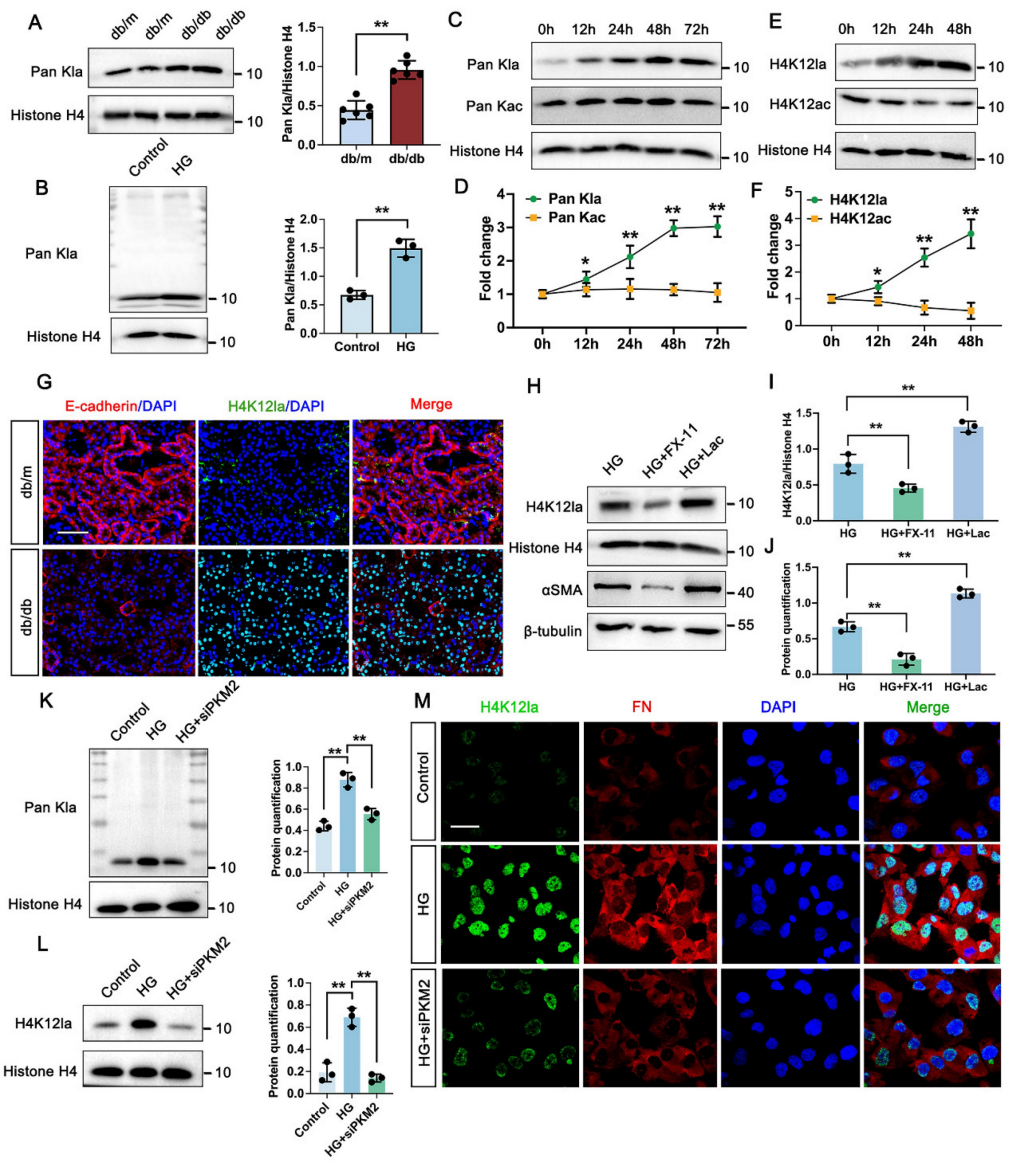
** PKM2 mediated lactate accumulation triggers histone lactylation in vitro and vivo.** A. Protein levels of Pan Kla in db/m and db/db group by western blot, and their semi-quantitative analyses (n=6); B. Protein levels of Pan Kla in Control and HG group by western blot, and its semi-quantitative analysis (n=3); C-D. The Pan Kla immunoblots of HK-2 cells at 0, 12, 24, 48 and 72 hours (h) treated with HG, and its semi-quantitative analysis (n=3); E-F. The H4K12la and H4K12ac immunoblots of HK-2 cells at 0, 12, 24 and 48 hours (h) treated with HG, and their semi-quantitative analyses (n=3); G. Immunostained for E-cadherin (red) and H4K12la (green), and counterstained with DAPI (blue) by IF staining in db/m and db/db group (n=6), scale bar=50 μm; H-J. The protein levels of H4K12la and αSMA by western blot in HG, HG + FX-11 and HG + Lac group, and their semi-quantitative analyses (n=3); K. Protein levels of Pan Kla in Control, HG and HG + siPKM2 group by western blot, and its semi-quantitative analysis (n=3); L. Protein levels of H4K12la in Control, HG and HG + siPKM2 group by western blot, and its semi-quantitative analysis (n=3); M. Double immunostained FN (red) and H4K12la (green), and counterstained with DAPI (blue) by IF staining in HK-2 cells in Control, HG and HG + siPKM2 group, scale bar=50 μm (n=3). Data represent the mean ± SD from three independent experiments. **P<.01 versus db/m (A) or Control group (B) by Student's t-test. *P<.05 or **P<.01 versus 0h (D, F) or HG group (I, J, K, L) by one-way ANOVA.

Considering that lactate functions as a metabolic substrate which can trigger histone lactylation 32, we hypothesized that this epigenetic modification might be altered in the kidneys of DKD models. As shown by Western blot analysis, histone proteins from db/db mice kidneys exhibited markedly elevated levels of pan-lysine lactylation (Pan-Kla) compared to those from db/m mice (Figure 2A). Similarly, global histone lactylation was significantly accumulated in high glucose (HG)-stimulated TECs relative to controls, with a prominent band appearing around 11 kDa, potentially corresponding to histone H4 (Figure 2B). Time-course immunoblotting of TECs further revealed that Pan-Kla levels were substantially upregulated from 12 to 48 hours and remained elevated up to 72 hours. However, the acetylation levels did not have markedly change within 72h (Figure 2C-D). Subsequent analysis of the specific histone modification H4K12la 30, revealed a comparable upregulation trend to global lactylation levels (Figure 2E-F). Supporting these findings, immunofluorescence staining of kidney sections from db/db mice demonstrated significantly increased H4K12la, with confocal microscopy indicating nuclear localization within the tubular compartment (Figure 2G). Subsequent Western blot analysis of H4K12la and αSMA proved an obvious elevation in TECs treated with lactate, however, downregulated in TECs treated with FX-11 compared with HG group (Figure 2H-J). Upon PKM2 knockdown, the signal of Pan Kla for histone was markedly decreased compared with HG group (Figure 2K). Further analysis revealed histone H4K12la exhibited remarkably decreased in HK-2 cells transfected with siPKM2 compared with HG group (Figure 2L). Moreover, in the siPKM2 group, minimal positive staining of H4K12la was observed in HK-2 cells (Figure 2M). Taken together, these findings indicate that tubular PKM2-mediated lactate accumulation stimulates fibrosis, possibly through the promotion of H4K12la.

### 3.4 Identification of potential downstream targets of H4K12la regulation

**Figure 3:**
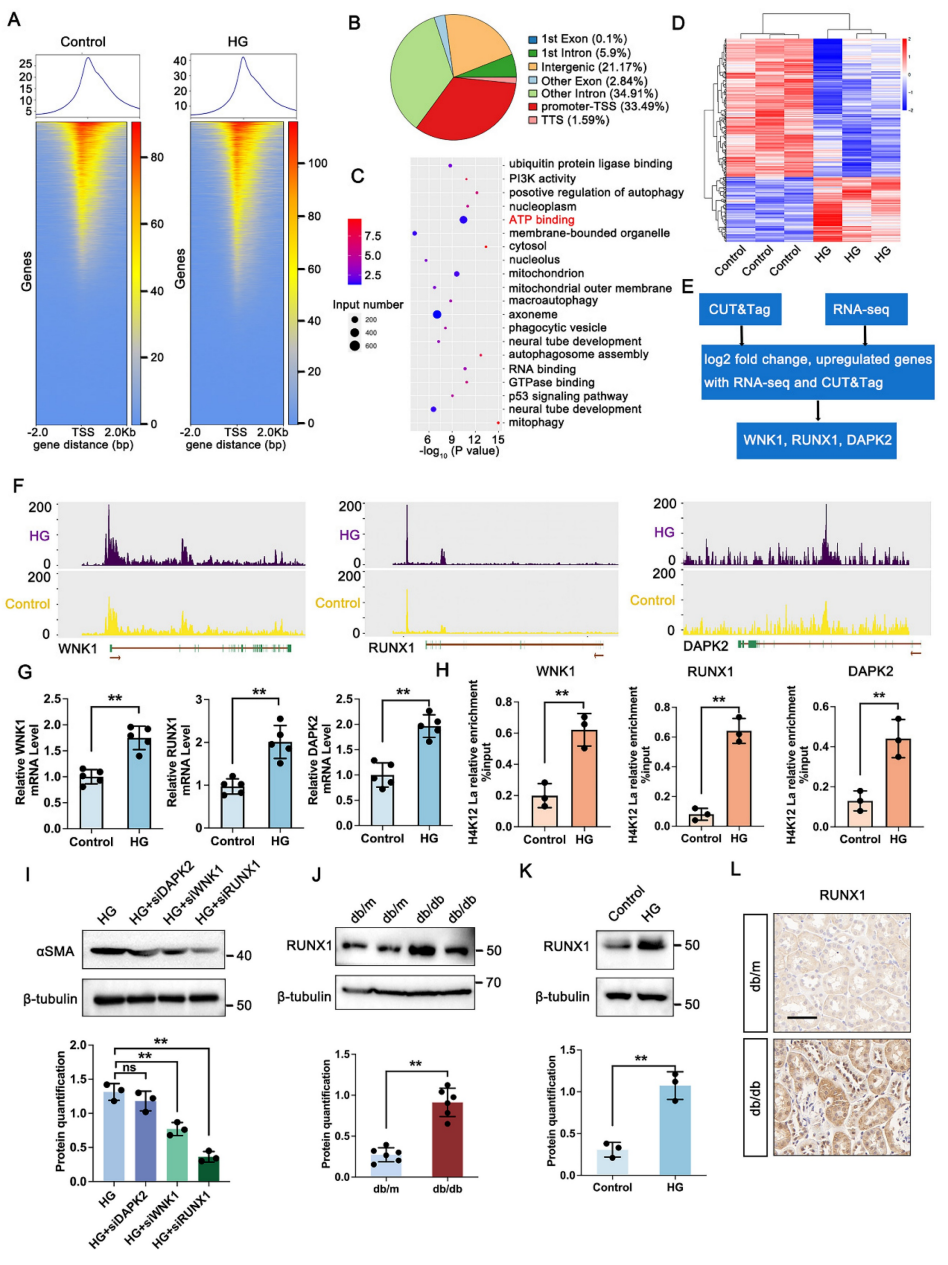
** H4K12la initiates RUNX1 transcription under HG condition in HK-2 cells.** A. Heatmaps for H4K12la binding peaks in HK-2 cells from Control and HG group. The red bar indicates the signal intensity of CUT&Tag; B. Pie plot of the different genomic distribution of H4K12la peaks between Control and HG group; C. Bubble chart showing the GO pathway enrichment analysis of upregulated genes with increased H4K12la modification; D. Heat map of the RNA-seq data from HK-2 cells in Control and HG group (n=3); E. Bioinformatics analysis filtered WNK1, RUNX1 and DAPK2 as downstream targets of H4K12la; F. IGV tracks for WNK1, RUNX1, and DAPK2 from CUT&Tag analysis; G. The mRNA level of WNK1, RUNX1 and DAPK2 in the Control and HG group in HK-2 cells by RT-qPCR (n=5); H. H4K12la occupancy analysis of WNK1, RUNX1 and DAPK2 in the Control and HG group in HK-2 cells by CUT&Tag-qPCR (n=3); I. Protein level of αSMA by western blot in the HG, HG transfected with siDAPK2, siWNK1 and siRUNX1 plasmid group in HK-2 cells, and its semi-quantitative analysis (n=3); J. Protein level of RUNX1 by western blot in the db/m and db/db group, and its semi-quantitative analysis (n=6); K. Protein level of RUNX1 by western blot in the Control and HG group in HK-2 cells, and its semi-quantitative analysis (n=3); L. The expression of RUNX1 in the db/m and db/db group by IHC assay, and its semi-quantitative analysis (n=6), scale bar=50 μm. Data represent the mean ± SD from three independent experiments. Data represent the mean ± SD from three independent experiments. **P<.01versus Control group (K) or db/m (G, H, J) by Student's t-test. **P<.01 versus HG group (I) by one-way ANOVA.

To explore downstream targets of histone lactylation in DKD, CUT&Tag analysis with anti-H4K12la antibodies was conducted on TECs cultured under control or HG conditions. HG exposure led to a marked increase in H4K12la binding peaks, particularly around TSS regions (Figure 3A), with 33.49% of differential peaks enriched in gene promoters (Figure 3B). These genes were predominantly associated with ATP-binding pathways (Figure 3C). To clarify the target genes of H4K12la, we further performed RNA sequencing of TECs treated with or without HG (Figure 3D). The heatmap derived from RNA-seq analysis clearly demonstrated differential expression of several key genes impacted in the ATP-binding pathway. Subsequent integrative analysis of CUT&Tag and RNA-seq data enabled the identification of WNK1, RUNX1, and DAPK1 as candidate target genes regulated by histone lactylation (Figure 3E). A genomic snapshot revealed the H4K12la modification sites in WNK1, RUNX1 and DAPK1 (Figure 3F). To validate the target genes of CUT&Tag and RNA-seq data, we performed RT-qPCR and ChIP-qPCR analysis to demonstrate the increased expression and H4K12la enrichment in the promoter regions of WNK1, RUNX1 and DAPK1. Notably, all three genes showed significant H4K12la modification levels (Figure 3G-H).

To evaluate whether these genes play a causal role in DKD-associated renal fibrosis, we generated knockdown models of DAPK2, WNK1, and RUNX1 in HG-exposed HK-2 cells via siRNA transfection. Silencing RUNX1 significantly reduced fibrosis-related markers, as shown by immunoblotting (Figure 3I). Further western blot analysis exhibited a marked upregulation in RUNX1 expression in HG-stimulated TECs and db/db mice kidneys (Figure 3J-K). Furthermore, IHC staining showed increased expression of RUNX1 in tubular region of kidney in db/db mice (Figure 3L). Altogether, these data support the notion that, upon DKD condition, tubular increased level of H4K12la directly facilitates RUNX1 activation by epigenetic modulation.

### 3.5 RUNX1 mediated glycolysis activated fibroblasts in DKD

**Figure 4:**
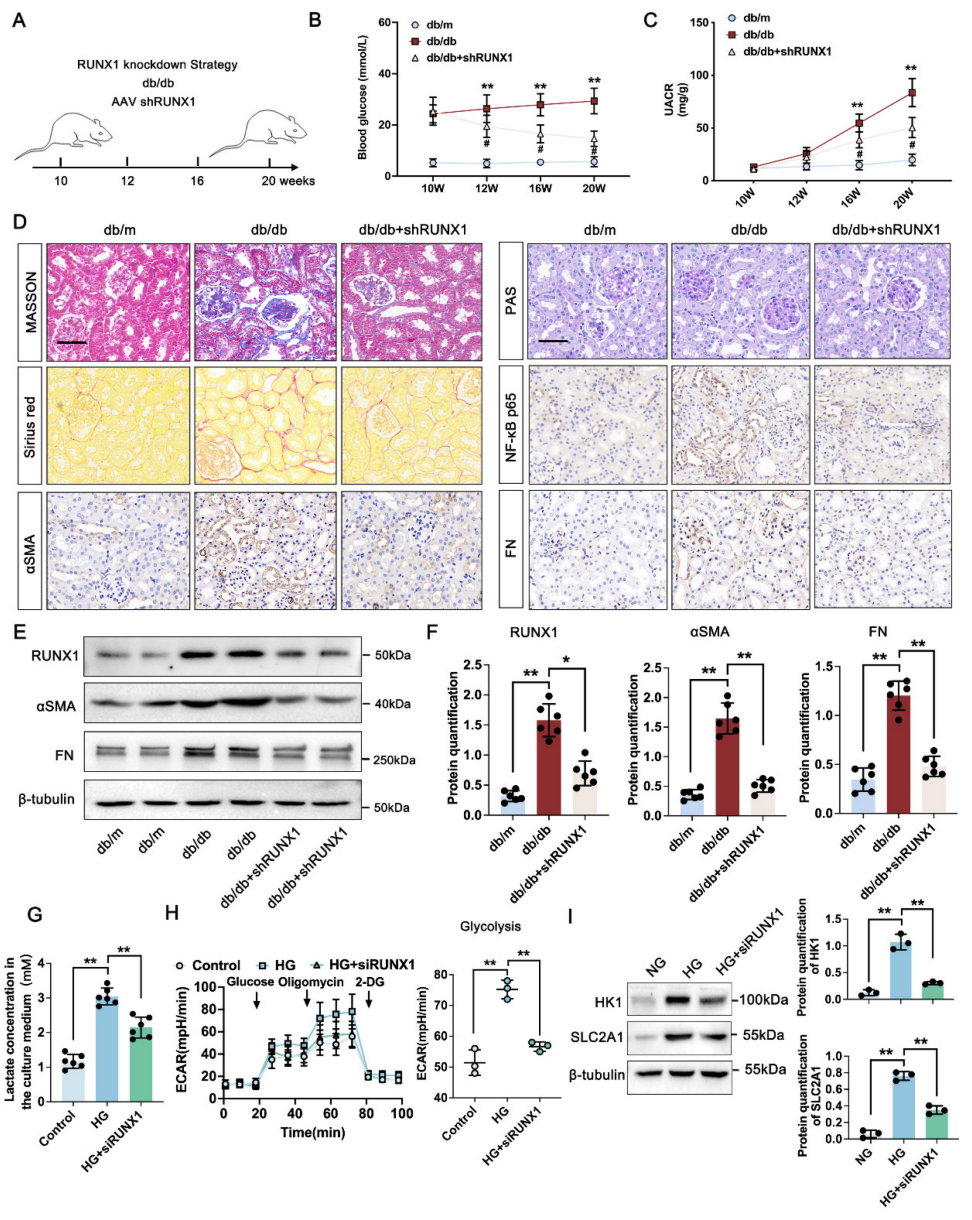
** Knockdown of RUNX1 inhibits TECs glycolysis and ameliorates renal fibrosis in DKD.** A. Schematic illustration of AAV9-shRUNX1 injection in the db/db group; B-C. Blood glucose levels Urinary albumin creatinine ratio (UACR) in db/m, db/db, and db/db + shRUNX1 group (n=6); D. Representative images of PAS, Masson, Sirius red staining and IHC staining (NF-κB p65, αSMA and FN) (scale bar=50 μm) in db/m, db/db, and db/db + shRUNX1groups (n=6); E-F. Protein levels of RUNX1, αSMA and FN in db/m, db/db, and db/db + shRUNX1 groups by western blot, with semi-quantitative analyses (n=6); G. The lactate concentration in HK-2 cells in the Control, HG and HG + siRUNX1 group (n=6); H. The ECAR levels after culturing with glucose followed by oligomycin and 2-DG in Control, HG and HG + siRUNX1 group (n=3); I. Protein levels of HK1 and SLC2A1 in Control, HG and HG + siRUNX1 group (n=3). Data represent the mean ± SD from three independent experiments.^ #^*P*<0.05 versus db/m, and **P<.01 versus db/db group or HG by one-way or two-way ANOVA.

To investigate the functional role of RUNX1 in DKD-related fibrosis, we produced a kidney-specific RUNX1 knockdown (RUNX1-KO) model using AAV9-shRNA delivery via in situ renal injection in db/db and db/m mice (Figure 4A). RUNX1 knockdown significantly lowered blood glucose levels starting at 12 weeks and reduced the UACR at 16 weeks in comparison with the db/db group (Figure 4B-C). Histological analyses revealed that diabetic mice developed pronounced glomerular and tubular abnormalities, including glomerulomegaly, mesangial matrix expansion, and tubular atrophy. These lesions were significantly mitigated by AAV-shRUNX1 administration. As opposed to db/m controls, db/db mice showed extensive renal fibrosis, which was effectively reduced by shRUNX1 as demonstrated by Masson, PAS, and Sirius Red staining (Figure 4D). IHC analysis confirmed that NF-κB p65, FN, and α-SMA levels were markedly elevated in db/db mice, but significantly suppressed in the shRUNX1-treated group (Figure 4D). Western blot analysis further validated that the expression levels of RUNX1, fibronectin (FN) and α-SMA were significantly elevated in the db/db group compared to the db/m controls. Notably, treatment with shRUNX1 markedly suppressed the expression of RUNX1, FN and α-SMA relative to the untreated db/db group (Figure 4E-F). To determine the connection among PKM2, renal fibrosis, and glycolysis, we conducted in vitro experiments. The cellular lactate level was significantly increased in TECs exposed to HG, whereas dramatically reduced upon RUNX1 depletion following HG condition (Figure 4G). Similarly, the glycolysis level of TECs was significantly lower in RUNX1-silence cells exposed to HG (Figure 4H). Previous study has found that RUNX1, as a transcription factor, promoted aerobic glycolysis through regulating SLC2A1 and HK1 expression 33. To identify potential glycolysis markers associated with RUNX1 in TECs, RUNX1 knockdown suppressed HK1 and SLC2A1 expression in TECs (Figure 4I). These results indicate that RUNX1 stimulates glycolysis in TECs participates in DKD by promoting fibrotic process.

### 3.6 H4K12la initiates RUNX1 expression contributes to positive feedback loop of glycolysis in DKD

**Figure 5:**
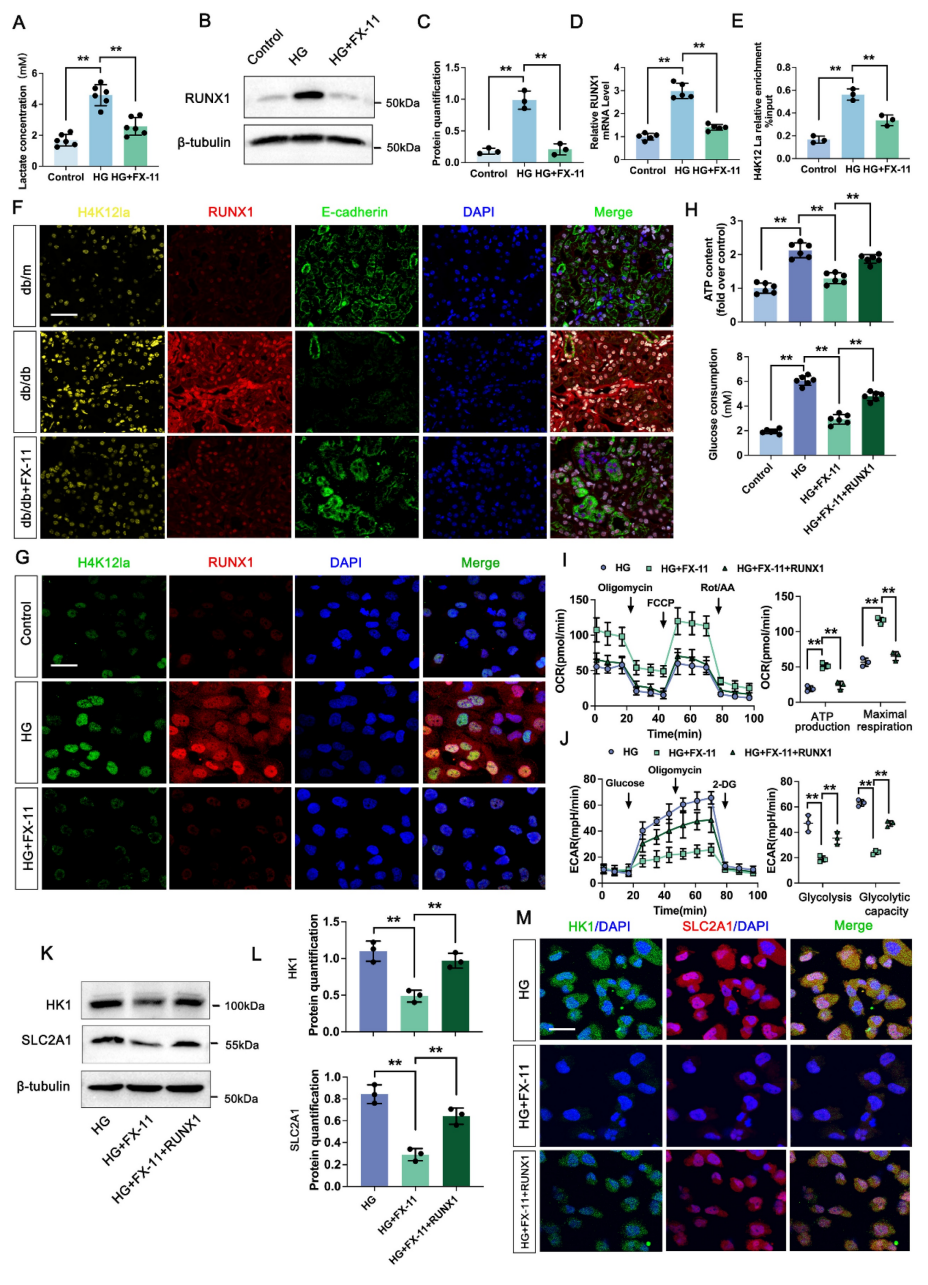
** Lactate-mediated elevation of H4K12la initiates RUNX1/glycolysis pathway.** A. The lactate concentration in HK-2 cells in the Control, HG and HG + FX-11 group (n=6); B-C. Protein levels of RUNX1 in Control, HG and HG + FX-11 group in HK-2 cells by western blot, with semi-quantitative analyses (n=3); D. The mRNA level of RUNX1 in the Control, HG and HG + FX-11 group in HK-2 cells by RT-qPCR (n=5); E. H4K12la occupancy analysis of RUNX1 in the Control, HG and HG + FX-11 group in HK-2 cells by CUT&Tag-qPCR (n=3); F. Immunostained for H4K12la (yellow), RUNX1 (red) and E-cadherin (green), counterstained with DAPI (blue) in db/m, db/db, db/db + FX-11 groups (scale bar=50 μm); G. Double immunostained RUNX1 (red) and H4K12la (green), and counterstained with DAPI (blue) by IF staining in HK-2 cells in Control, HG and HG + FX-11 group, scale bar=50 μm (n=3); H. ATP content and glucose consumption in Control, HG, HG + FX-11 and HG + FX-11 + RUNX1 group in HK-2 cells (n=6); I. Mitochondrial oxidative capacity in HK-2 cells in the Control, HG, HG + FX-11 and HG + FX-11 + RUNX1 group (n=3); J. The ECAR levels after culturing with glucose followed by oligomycin and 2-DG in Control, HG, HG + FX-11 and HG + FX-11 + RUNX1 group (n=3); K-L. Protein levels of HK1 and SLC2A1 in HK-2 cells in the Control, HG, HG + FX-11 and HG + FX-11 + RUNX1 group by western blot, with semi-quantitative analyses (n=3); M. Double immunostained SLC2A1 (red) and HK1 (green), and counterstained with DAPI (blue) by IF staining in HK-2 cells in HG, HG + FX-11 and HG + FX-11 + RUNX1 group, scale bar=50 μm (n=3). Data represent the mean ± SD from three independent experiments. **P<.01 versus HG group (A, C, D, E, H) or HG + FX-11 group (H, I, J, L) by one-way ANOVA.

We next analyzed the effect of H4K12la/RUNX1 on TECs function in DKD. FX-11 significantly suppressed lactate accumulation of TECs compared with HG group (Figure 5A). Western blot analysis showed that FX-11 exhibited significant inhibition effect on RUNX1 expression (Figure 5B-C). ChIP-qPCR implied a decrease in H4K12la enrichment in the promoter regions of RUNX1, and RT-qPCR results validated downregulated target gene expression after FX-11 treatment (Figure 5D-E). Triple IF staining of H4K12la (Yellow), RUNX1 (Red) and E-cadherin (Red), indicating renal tubule area in db/db group presented significantly higher fluorescent intensities of H4K12la and RUNX1, but reversed in the FX-11-treated group (Figure 5F). Similarly, the alteration of H4K12la and RUNX1 of the HK-2 cells in FX-11 treated group was further supported by using double IF staining (Figure 5G). To further confirm the glycolysis changes induced by H4K12la/RUNX1 in TECs under DKD condition, we applied RUNX1 over-expression plasmid transfect to HK-2 cells co-cultured with FX-11 under HG condition. As expected, ATP content and glucose consumption in the supernatant of HK-2 cells were higher in the HG-stimulated group than in the control group, FX-11 treatment reversed ATP production and glucose consumption compared with the HG group, however RUNX1 over-expression abolished the function of FX-11 (Figure 5H). Furthermore, key metabolic parameters of HK-2 cells were assessed using an XF^24^ extracellular flux analyzer. FX-11 treatment resulted in reduced glycolytic capacity while enhancing mitochondrial respiration, including ATP-coupled and maximal respiration, compared to the high glucose (HG) group. Notably, these FX-11-induced metabolic changes were reversed upon RUNX1 overexpression, with glycolytic capacity and mitochondrial respiration restored to levels comparable to those in the HG group (Figure 5I-J). Next, we assessed the function of H4K12la/RUNX1 on glycolysis related genes under DKD condition. As illustrated in Figure 5K-L, western blot analysis validated that the expression levels of HK1 and SLC2A1, which were suppressed by FX-11 treatment, were restored upon transfection with a RUNX1 overexpression plasmid in HK-2 cells. Moreover, this trend was recapitulated by the double IF staining of SLC2A1 (Red) and HK1 (Green) in Figure 5M. Our results propose the presence of a positive feedback loop in which RUNX1 upregulates HK1 and SLC2A1 expression and increases lactate levels, which in turn enhances RUNX1 expression via elevation of H4K12la levels.

### 3.7 SIRT3 is the deacyltransferase for histone lactylation of RUNX1 in DKD

**Figure 6:**
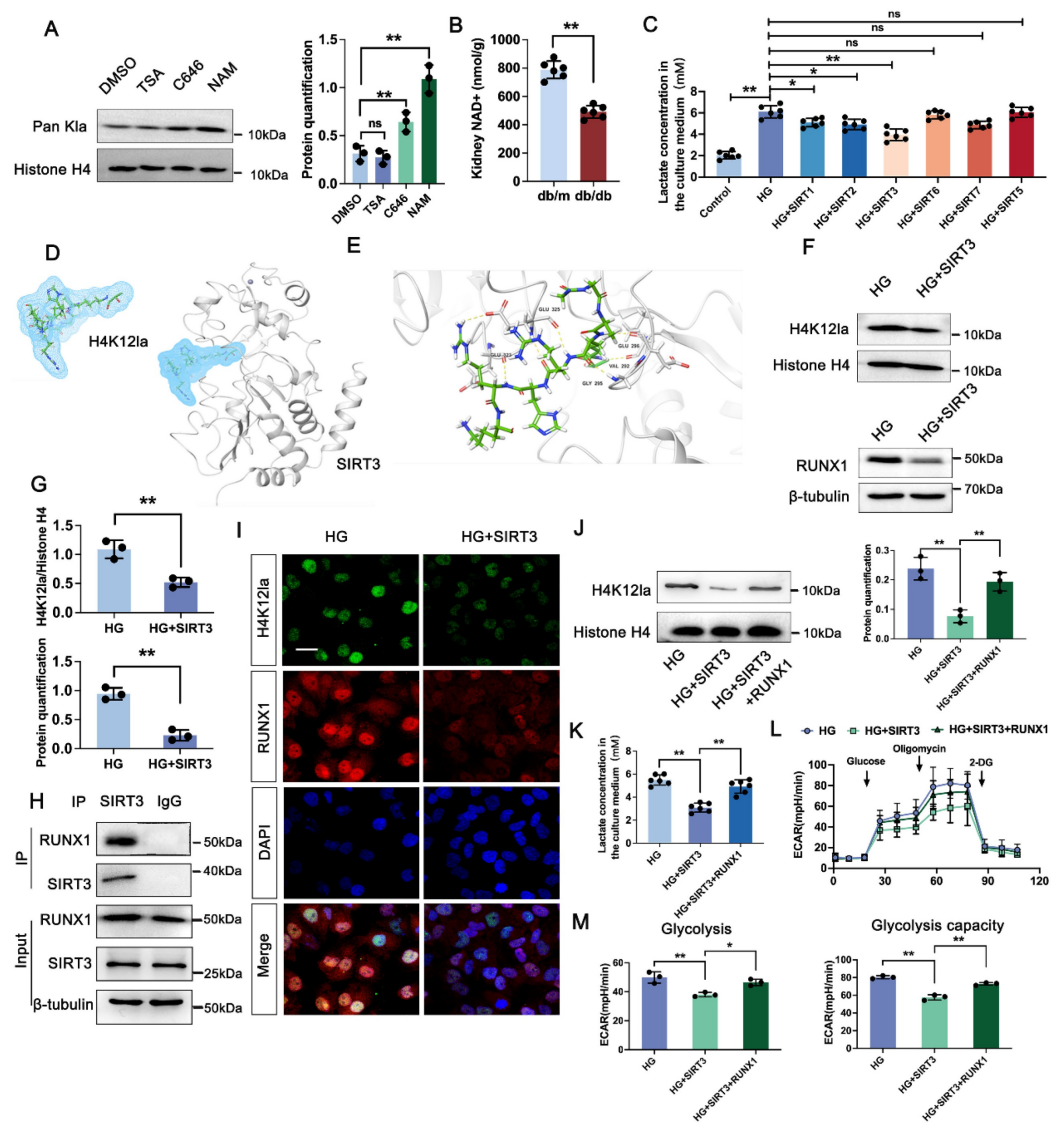
** SIRT3-mediated deacetylation of RUNX1 modulates histone lactylation in DKD.** A. Protein levels of Pan Kla in HK-2 cells in DMSO, TSA, C646 and NAM group by western blot, and their semi-quantitative analyses (n=3); B. Bargraph representing NAD+ levels in db/db and db/m group (n=6); C. The lactate concentration in HK-2 cells in the Control, HG and HG transfected with SIRT1, SIRT2, SIRT3, SIRT5, SIRT6 and SIRT7 group (n=6); D. The overall structures of SIRT3 (white)-H4K12la (green) complex structure; E. Autodock vina 1.1.2 was performed to analyze the binding mode between peptide and SIRT3; F-G. Protein levels of H4K12la and RUNX1 in HK-2 cells in HG and HG+ SIRT3 group by western blot, and their semi-quantitative analyses (n=3); H. HK-2 cells were lysed and immunoprecipitated using anti-SIRT3 antibody or control IgG, followed by detection of RUNX1; I. Double immunostained RUNX1 (red) and H4K12la (green), and counterstained with DAPI (blue) by IF staining in HK-2 cells in HG and HG + SIRT3 group, scale bar=50 μm (n=3); J. Protein level of H4K12la by western blot in the HG, HG + SIRT3 and HG +SIRT3 + RUNX1 group in HK-2 cells, and its semi-quantitative analysis (n=3); K. The lactate concentration in HK-2 cells in the HG, HG + SIRT3 and HG +SIRT3 + RUNX1 group (n=6); L-M. The ECAR levels after culturing with glucose followed by oligomycin and 2-DG in the HG, HG + SIRT3 and HG +SIRT3 + RUNX1 group (n=3). Data represent the mean ± SD from three independent experiments. **P<.01 versus DMSO group (A) or HG group (C) or HG + SIRT3 group (J, K, M) by one-way ANOVA, **P<.01versus HG group (G) or db/m group (B) by Student's t-test.

We next determined the enzymatic mechanism of lactylation. In Figure 6A, western blot analysis showed both HDAC inhibitor (TSA) and P300 inhibitor (C646) had no significant change on the overall histone lactylation level, but Sirtuins inhibitor (NAM) caused an increase in the overall histone lactation level. Sirtuins, which is well known to be a nicotinamide adenine dinucleotide+ (NAD+)-dependent protein deacetylase. NAD+ levels were detected by the NAD+ assay kit, revealing a significant decrease in NAD+ levels in db/db group compared to db/m group (Figure 6B). In comparison to the negative HG group, HK-2 cells transfected with the SIRT5-7 overexpression plasmid exhibited minimal changes of lactate level. However, SIRT1-3 over-expressed group showed a certain decrease of lactate level compared with HG group, and Sirt3 showed a higher activity on lactate production (Figure 6C). A molecular docking approach was employed to evaluate the binding conformation between SIRT3 and H4K12la, aiming to elucidate the catalytic function of SIRT3 in histone lactylation regulation during DKD (Figure 6D-E). In line with the aforementioned findings, overexpression of SIRT3 reduced the levels of H4K12la and RUNX1 (Figure 6F-G). We then verified the SIRT3-RUNX1 interaction by coimmunoprecipitation (Co-IP) in vitro (Figure 6H). H4K12la and SIRT3 were found to colocalize in the nuclei of HG-treated HK-2 cells, as shown by immunofluorescence staining. SIRT3 overexpression reduced both H4K12la and RUNX1 expression compared with the HG group (Figure 6I). We further determine whether SIRT3 affect glycolysis through RUNX1 expression. Our data indicated that overexpression of SIRT3 downregulated H4K12la expression, whereas, increased RUNX1 expression promoted H4K12la compared with the SIRT3 group (Figure 6J). Moreover, SIRT3 inhibited the lactate accumulation, decreased glycolysis and glycolysis capacity levels, RUNX1 overexpression reversed SIRT3 induced the promotion of glycolysis (Figure 6K-M). These data collectively indicate the SIRT3-induced H4K12la in the development of DKD.

### 3.8 SIRT3 downregulation is involved in the pathogenesis of DKD by triggering RUNX1 mediated glycolysis

**Figure 7:**
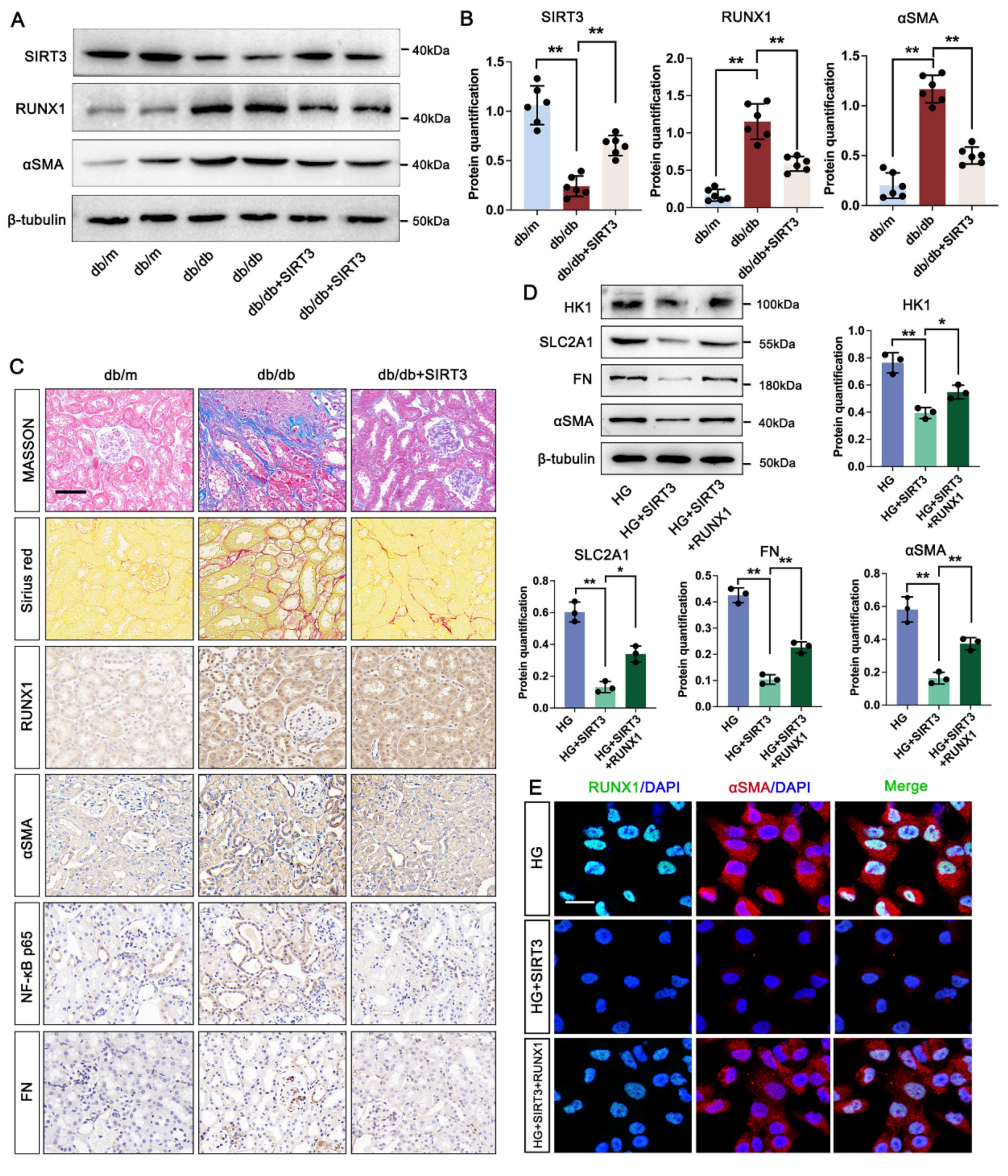
** SIRT3 increased RUNX1 mediated glycolysis and promotes renal fibrosis in DKD.** A-B. Protein levels of SIRTS, RUNX1 and αSMA in db/m, db/db, and db/db + SIRT3 groups by western blot, with semi-quantitative analyses (n=6); C. Representative images of Masson, Sirius red staining and IHC staining (RUNX1, NF-κB p65, αSMA and FN) (scale bar=50 μm) in db/m, db/db, and db/db + sSIRT3 groups (n=6); D. Protein level of HK1, SLC2A1, FN and αSMA by western blot in the HG, HG + SIRT3 and HG + SIRT3 + RUNX1 group in HK-2 cells, and its semi-quantitative analysis (n=3); E. Double immunostained αSMA (red) and RUNX1 (green), and counterstained with DAPI (blue) by IF staining in HK-2 cells in HG, HG + SIRT3 and HG +SIRT3 + RUNX1 group, scale bar=50 μm (n=3). Data represent the mean ± SD from three independent experiments. *P<.05 or **P<.01 versus db/db group (B) or HG +SIRT3 group (D) by one-way ANOVA.

Firstly, our data indicated that the level of SIRT3 was increased in the kidney of DKD mice. We then used AAV9-Sirt3 overexpression mice and further explore the function of SIRT3 in DKD. Compared with db/db group, overexpression of SIRT3 decreased the expression of RUNX1 and αSMA (Figure 7A-B). Moreover, overexpression of SIRT3 presented less damage to the kidney fibrotic structural by MASSON and Sirius Red staining, and reduced collagen fiber deposition by IHC staining for FN and αSMA. In addition, we found that overexpression of SIRT3 leads to a decrease in the expression of RUNX1 and inflammatory mediator (NF-κB p65) (Figure 7C). To further identified the mechanism of SIRT3 in TECs, a RUNX1-overexpression and co-transfected with SIRT3-overexpression HK-2 cell line was established. Western blot analysis showed SIRT3 significantly decreased the expression of fibrosis-related protein (FN and αSMA), and glycolysis-related gene (HK1 and SLC2A1) under the stimulation of HG in vitro, whereas overexpression of RUNX1 abolished the protective function of SIRT3 on fibrotic process and glycolysis pathway (Figure 7D). In addition, the immunofluorescence staining of RUNX1 and αSMA showed that SIRT3 significantly decreased the level of RUNX1 and αSMA, while overexpression of RUNX1 increased the level of RUNX1 and αSMA (Figure 7E). These data indicate that SIRT3 deficiency-induced kidney fibrosis participates in DKD by triggering RUNX1 mediated glycolysis process.

## 4. Discussion

Despite notable advancements in the understanding of DKD pathogenesis and detection markers of DKD, it continues to propose a significant threat as a common complication of critical illness 34-36. An increasing body of evidence shows that metabolic reprogramming within renal tubules plays a pivotal role in driving renal fibrosis during DKD progression 37, 38. The main finding of the present study was that SIRT3 induced H4K12la promoted glycolysis, thus participating the fibrotic process during DKD. Moreover, SIRT3 interacted with RUNX1 to trigger glycolysis in DKD via promoted the transcription of HK1 and SKC2A1. Notably, inducing SIRT3-mediated lactylation effectively attenuated renal fibrosis, thus ameliorating renal dysfunction in DKD. These findings uncover a novel mechanism whereby tubular glycolytic dysfunction promotes renal fibrosis through epigenetic regulation and SITR3/RUNX1 pathway activation.

Pyruvate kinase M2 (PKM2) is a crucial enzyme in the glycolysis pathway that controls the rate at which phosphopyruvate is converted into pyruvate and ATP by phosphorylation 39, 40. Previous study has found that the expression of PKM2 was significantly increased in both in vivo and in vitro models, and the knockdown of PKM2 attenuated CaOx crystal-induced renal fibrosis 20. Additionally, recent study has indicated that empagliflozin reduced PKM2 expression, thereby alleviating EMT and renal fibrosis 41. In the present study, it was found that the expression of PKM2 was significantly increased in tubular region by ST-analysis data, and the knockdown of PKM2 attenuated renal fibrosis in DKD. Lactate acts as a crucial signaling molecule, regulating immune cell activity and contributes to microenvironmental remodeling in multiple disease contexts through its effects on cell signaling pathways and transcriptional regulation 13, 42, 43. Diabetic renal tissues show a metabolic shift characterized by increased LDHA and lactate levels, alongside diminished ATP production. Emerging evidence indicates a strong link between circulating and urinary levels of glucose and lactate in individuals with diabetes. Notably, proximal tubular cells contribute to this lactate production, which is diminished by pharmacological inhibition of sodium-glucose cotransporter-2 (SGLT2) 44. These findings preliminarily confirm that lactate accumulation is implicated in the occurrence and development of DKD. In the course of this research, the lactate level in the urine of DKD patients is increased, which is negatively correlated with eGFR. Lactate administration exacerbates renal pathological damage and interstitial fibrosis in diabetes mice, whereas inhibiting lactic acid production can significantly alleviate renal fibrosis. These studies connect renal function to energy metabolism and suggest that targeting cellular energy pathways could offer a novel approach for developing DKD therapies.

Of note, several studies have highlighted the interplay between energy metabolism and histone modifications. Like other chromatin modifications, lactylation can remodel histone architecture, thereby affecting chromatin accessibility and transcriptional activity. Importantly, lactylation levels are directly influenced by the spatial distribution of lactate, forming a functional bridge between cellular metabolism and epigenetic control 45-47. Histone H4K12 lactylation was found to be markedly increased in a manner dependent on lactate availability, as observed in both diabetic mice and in vitro experiments. Integrated CUT&Tag with RNA-seq revealed that H4K12la localized at RUNX1 promoter areas, enhancing transcriptional activation and amplifying subsequent glycolytic signaling. Besides, the study here found that PKM2 functions as a positive regulator in controlling histone lactylation, which ultimately activates RUNX1 in renal tubular cells during DKD. However, the evidence regarding the pathogenesis of DKD and lactylation association with RUNX1 remains insufficient.

By defining the positive feedback loop, we demonstrate that H4K12la subsequently enhances the expression of RUNX1-regulated glycolytic genes. Further investigation into the potential interplay between histone acetylation and lactylation, and their regulatory effects on gene expression in DKD development. Despite existing studies, the specific function of RUNX1 in DKD has yet to be fully elucidated. The role of RUNX1 in diseases exhibits significant tissue specificity and environmental dependence. RUNX1 is frequently mutated in estrogen receptor-positive luminal breast cancer, a finding that supports the notion of the disease as a stem cell-associated disorder 48, 49. While in metabolic and diseases, it is closely associated with epigenetic modifications 50, 51. RUNX1 has been recognized as a key mediator of inflammatory responses and fibrotic progression in AKI. For example, RUNX1 facilitates the transcription of pro-inflammatory cytokines like IL-6 by interacting with the NF-κB pathway, exacerbating tubular injury and interstitial inflammation 52. Our study discovered that the upregulated RUNX1 expression in tubular region of kidney during DKD. Since the CUT&Tag and RNA-seq data here has shown that RUNX1 was involved in the ATP binding pathway. miR-30d has been shown to restrain aerobic glycolysis by downregulating SLC2A1 and HK1 expression via direct targeting of RUNX1, a transcription factor that binds to the promoters of these genes 33. Furthermore, for the first time, we reveal that RUNX1 ameliorates renal fibrotic progression in DKD through glycolysis. Our study shows that FX-11 inhibits H4K12la-mediated RUNX1 expression, thus inhibits the level of HK1 and SCL2A1, accompany with the lower glycolytic capacity and increased ATP production compared with the HG group. Over expression with RUNX1 plasmid abolished the function of FX-11 on RUNX-mediated glycolysis pathway, implying that modulation of this specific epigenetic modification may provide new therapeutic avenues for the treatment of DKD.

In histone modification, to precisely regulate the "histone code", a series of enzymes to generate, eliminate, or recognize these post-translational modifications. When histone sites with differential modifications are identified, relevant transcription factors are recruited to specific positions in the genome to regulate the expression of related genes 53, 54. It has been reported that histone lactylation is primarily regulated by the availability of lactyl-CoA and the catalytic action of specific enzymes, notably P300/CBP, which serve as major writers of lactylation marks in macrophages 55. Moreover, members of the HDAC1-3 and Sirtuins families can not only remove acetyl groups from lysine residues but also eliminate lactylation modification groups from the ε-amino groups of histone lysines, thereby exerting a de-lactylation effect 32, 46. However, it remains unknown whether histone regulatory enzymes can participate in histone lactylation modification in TECs during DKD. Our study extended the analysis to assess the enzymatic activity of P300, HDACs, and several Sirtuins (SIRT1-3, 5-7) in the context of histone lactylation, revealing a significant role for SIRT3 in mediating this process in TECs. It has been reported that SIRT3 removes lactylation modifications from critical mitochondrial enzymes such as PDHA1 and CPT2, leading to their reactivation and subsequent enhancement of oxidative phosphorylation 56. We found that SIRT3 overexpression in HK-2 cells suppressed both H4K12la levels and RUNX1 expression. Furthermore, a direct interaction between SIRT3 and RUNX1 was observed, suggesting for the first time that SIRT3 may act as a histone lactylation eraser targeting RUNX1.

Notably, lactate levels elevated during DKD were significantly reduced upon SIRT3 overexpression, and SIRT3-reduced H4K12la was reversed by overexpression of RUNX1, suggesting that SIRT3 can regulate H4K12la through modulating lactate levels. Concurrently, SIRT3 loss promotes RUNX1 transcription, actives glycolysis-mediated renal fibrotic process. Finally, SIRT3 overexpression attenuated fibroblast activation, indicating that SIRT3 alleviates fibrosis through H4K12la/RUNX1 feedback loop.

Although our study elucidated the impact of RUNX1 H4K12 lactylation on glycolysis both in vivo and in vitro, certain limitations should be acknowledged. Firstly, the human kidney samples we collected were obtained from the distal normal tissue of cancer patients. Although they were histologically confirmed to be composed of normal cells, the availability of human aging tissue samples remained a limitation of this study. Secondly, our investigation did not assess lactylation of non-histone proteins, which may exert more precise and direct regulatory effects on the function of tubular epithelial cells (TECs). Future research incorporating mass spectrometry and site-directed mutagenesis will be essential to clarify the functional significance of lactylation on specific monocyte-associated proteins.

## 5. Conclusion

In conclusion, our study demonstrates that a novel SIRT3/H4K12la/RUNX1 axis that drives renal fibrosis in DKD. Under DKD conditions, SIRT3 expression is downregulated, leading to H4K12la accumulation in TECs that directly activates RUNX1 expression, thus promoting fibroblast activation. As demonstrated by SIRT3 overexpression experiments showing concurrent reductions in H4K12la levels, lactate accumulation, and fibrotic marker expression. This study establishes SIRT3 may serve as a potential therapeutic target against DKD-induced renal fibrosis.

## Supplementary Material

Supplementary figures and tables.
