# Supplementary material for: Delactylase effects of SIRT3 on a positive feedback loop involving the RUNX1-glycolysis-histone lactylation in diabetic kidney disease
Source: Int J Biol Sci. 2026 Jan 15;22(4):1775–92. doi: 10.7150/ijbs.126011 (PMC12905586; doi:10.7150/ijbs.126011)
Supplement: Supplementary file 1 — Supplementary figures and tables. [file ijbsv22p1775s1.pdf]

Figure S1. The expression of glycolysis related genes were decreased in both diabetic kidney and DKD patients

A. The protein levels of PKM2 and HK2 in DKD mice or DKD patients group by IHC assay (n=6), scale bar=50 μm; B. Immunostained E-cadherin (red) and PKM2 (green), and counterstained with DAPI (blue) by IF staining in db/m and db/db group (n=6), scale bar=50 μm.

Figure S2. **Supplemental lactate promotes renal fibrosis in db/db mice.**

A. Schematic illustration of lactate administration in the db/db group; B. The lactate concentration in the db/m, db/db, db/m+lactate (Lac) and db/db + lactate (Lac) group (n=8); C. Representative images of Masson staining, Sirius red and IHC staining (αSMA, Vimentin and FN) (scale bar=50 μm) in db/m, db/db, db/m + lactate and db/db + lactate (Lac) groups (n=6); D. Protein levels of αSMA, Vimentin and FN in db/m, db/db, db/m + lactate and db/db + lactate (Lac) groups by Western blot (n=6). Data represent the mean ± SD from three independent experiments. ***P*<.01 versus db/db group by one-way ANOVA. **P<.01versus db/m group (D) or Control group (F) by Student's t-test.

Figure S3. **Inhibition of lactate attenuates renal fibrosis in db/db mice.**

A. Illustrative overview of the FX-11 intervention strategy in db/db mice; B. The lactate concentration in the db/m, db/db, db/m + FX-11 and db/db + FX-11 groups (n=8); C. Representative images of Masson staining, Sirius red and IHC staining (αSMA, Vimentin and FN) (scale bar=50 μm) in db/m, db/db, db/m + FX-11 and db/db+FX-11 groups (n=6); D. Protein levels of αSMA, Vimentin and FN in db/m, db/db, db/m + FX-11 and db/db + FX-11 groups by Western blot (n=6). Data represent the mean ± SD from three independent experiments. ***P*<.01 versus db/db group by one-way ANOVA.

Figure S4. PKM2 deficiency alleviates renal fibrosis during DKD.

A. The lactate concentration in HK-2 cells in the Control, HG, HG + siPKM2 and HG + siHK2 group (n=3); B-C. Protein levels of FN and αSMA in Control, HG and HG + siPKM2 group in HK-2 cells by western blot, with semi-quantitative analyses (n=3); D. Schematic illustration of PKM2 deficiency in the db/db group; E. Representative images of Masson, Sirius red staining and IHC staining (αSMA and FN) (scale bar=50 μm) in db/m, db/db, and db/db + shPKM2 groups (n=6); F. Immunostained E-cadherin (red) and FN (green), and counterstained with DAPI (blue) by IF staining in db/m, db/db, and db/db + shPKM2 groups (n=6), scale bar=50 μm; G. Protein levels of FN and αSMA in db/m, db/db, and db/db + shPKM2 groups by western blot, with semi-quantitative analyses (n=6). Data are presented as mean ± standard deviation from three independent experiments. ***P*<.01 versus HG (A, B) or db/db group (G) by one-way ANOVA.

Figure S1

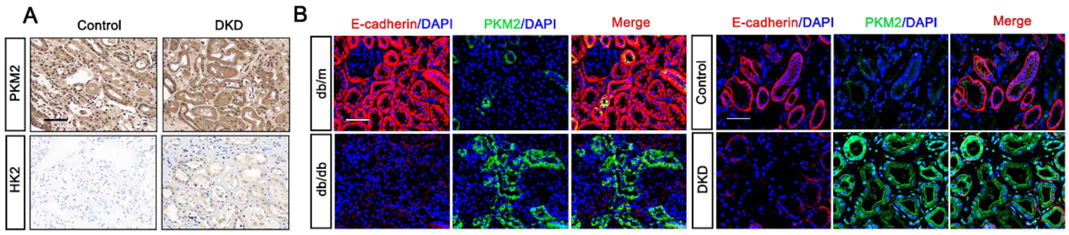
A
Control
DKD
PKM2
HK2
B
E-cadherin/DAPI
PKM2/DAPI
Merge
db/m
db/db
E-cadherin/DAPI
PKM2/DAPI
Merge
Control
DKD

Figure S2

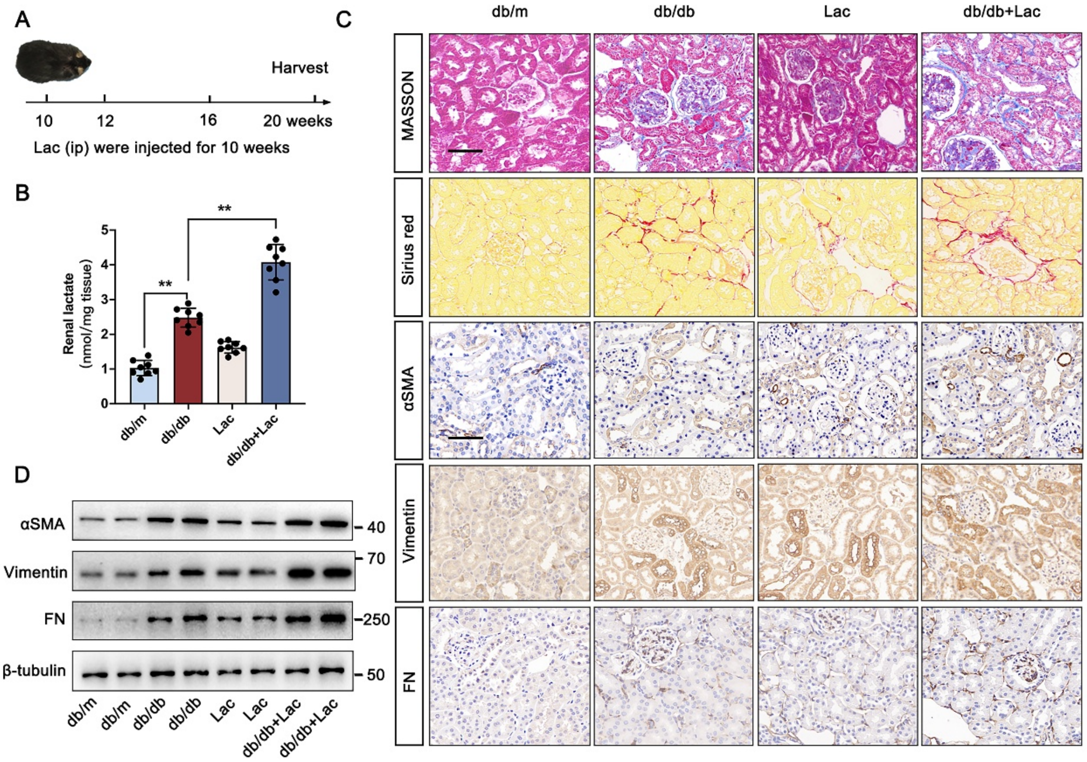
A
Harvest
10
12
16
20 weeks
Lac (ip) were injected for 10 weeks
B
Renal lactate (nmol/mg tissue)
**
**
db/m
db/db
Lac
db/db+Lac
C
db/m
db/db
Lac
db/db+Lac
MASSON
Sirius red
αSMA
Vimentin
FN
D
αSMA
Vimentin
FN
β-tubulin
40
70
250
50
db/m
db/m
db/db
db/db
Lac
Lac
db/db+Lac
db/db+Lac

Figure S3

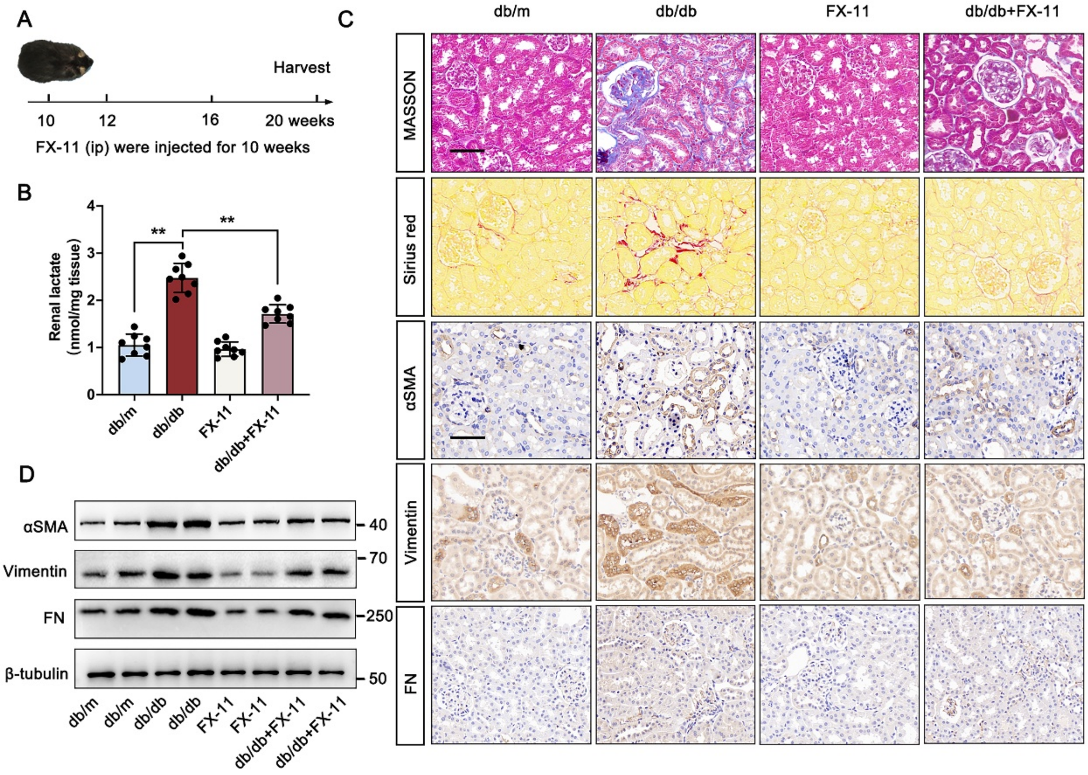
A
Harvest
10
12
16
20 weeks
FX-11 (ip) were injected for 10 weeks
B
Renal lactate (nmol/mg tissue)
**
**
db/m
db/db
FX-11
db/db+FX-11
C
db/m
db/db
FX-11
db/db+FX-11
MASSON
Sirius red
αSMA
Vimentin
FN
D
αSMA
Vimentin
FN
β-tubulin
40
70
250
50

Figure S4

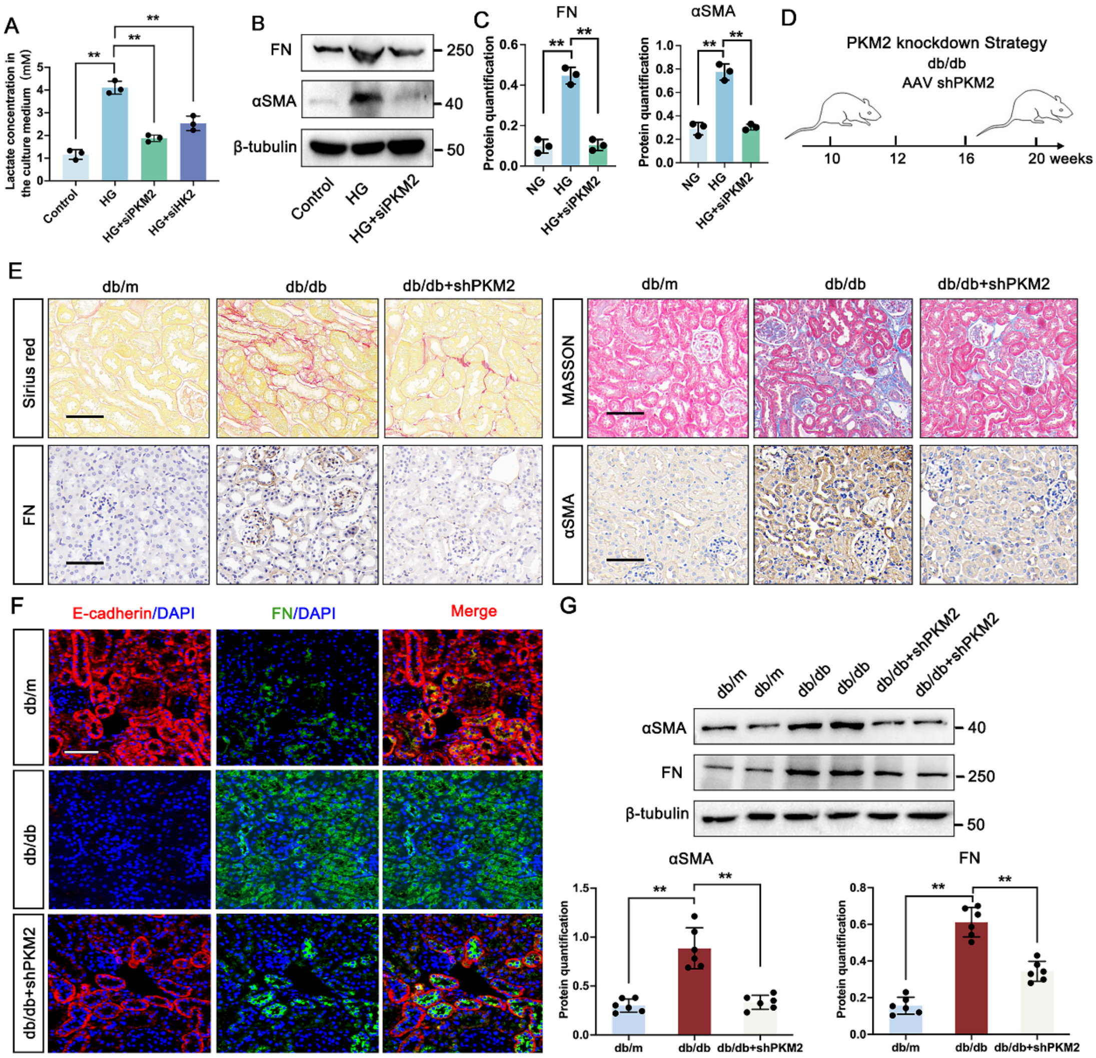

A
Lactate concentration in the culture medium (mM)
Control
HG
HG+siPKM2
HG+siHK2
B
FN
αSMA
β-tubulin
250
40
50
Control
HG
HG+siPKM2
C
FN
αSMA
Protein quantification
NG
HG
HG+siPKM2
D
PKM2 knockdown Strategy
db/db
AAV shPKM2
10
12
16
20 weeks
E
db/m
db/db
db/db+shPKM2
Sirius red
MASSON
FN
αSMA
F
E-cadherin/DAPI
FN/DAPI
Merge
db/m
db/db
db/db+shPKM2
G
db/m
db/db
db/db+shPKM2
αSMA
FN
β-tubulin
40
250
50
Protein quantification

Supplemental Table S1

Table S1. Primers for RT-qRCR.

Primer name		Sequence
Has-PKM2	Sense	ATGGCTGACACATTCCTGGAGC
	Anti-sense	CCTTCAACGTCTCCACTGATCG
Has-HK2	Sense	GAGTTTGACCTGGATGTGGTTGC
	Anti-sense	CCTCCATGTAGCAGGCATTGCT
Has-WNK1	Sense	CAGTGTGGAACCAGAGGGTGAT
	Anti-sense	TTTGCTGTGGCATGGAAGACGC
Has-RUNX1	Sense	CCACCTACCACAGAGCCATCAA
	Anti-sense	TTCACTGAGCCGCTCGGAAAAG
Hsa-DAPK2	Sense	GCACCACAATGTCATCACGCTG
	Anti-sense	CCTCACTCAGTGACTCCTTCTG
hsa-β-actin	Sense	CCTGGCACCCAGCACAAT
	Anti-sense	GCTGATCCACATCTGCTGGAA

Supplemental Table S2

Table S2. Primers for ChIP-qPCR.

Primer name		Sequence
Has-WNK1	Sense	GCGCGCGTTTCCTCTCAGT
	Anti-sense	CCAGCGGGGCTGAACTTCT
Has-RUNX1	Sense	AGGAGGCGGAGGAGGAGAG
	Anti-sense	TTCTGGGGAGGAGGAGCGT
Has-DAPK2	Sense	GCGCCTGCGGAGTGAGAGA
	Anti-sense	CCCACGCCCGTCTCCTTTA
